# The role of community health workers in influencing social connectedness using the household model: a qualitative case study from Malawi

**DOI:** 10.1080/16549716.2022.2090123

**Published:** 2022-08-12

**Authors:** Myness Kasanda Ndambo, Fabien Munyaneza, Moses Aron, Henry Makungwa, Basimenye Nhlema, Emilia Connolly

**Affiliations:** Community Health Department, Partners In Health/Abwenzi Pa Za Umoyo, Neno, Malawi

**Keywords:** Social connectedness, community health workers, household model, interaction, Neno

## Abstract

**Background:**

Community health workers (CHWs) play a vital role in facilitating social connectedness, building trust, decrease stigma, and link communities to essential healthcare and social support services. More studies are needed to understand the factors facilitating these interactions among CHWs, clients, and community members.

**Objective:**

This study examined the CHW role and relationships between CHWs, communities, and health facilities that promote trust, positive relationships, and social connectedness.

**Methods:**

In 2016, the CHW program in Neno District, Malawi, was transitioned to a household-level assignment of CHWs to provide screening, linkage to care, and psychosocial and chronic disease support from a disease-based program. We employed an exploratory qualitative study with thematic analysis linked to Fredrickson’s broaden-and-build theory of positive emotions through focus group discussions (FGDs) and in-depth interviews (IDIs) to understand the impact of the household assignment. We purposively sampled community stakeholders, CHWs, health service providers, and clients (total N = 180) from October 2018 through March 2020. All interviews were audiotaped, transcribed verbatim, translated, coded, and analyzed.

**Results:**

Participants reported decreased stigma and discrimination with increased trust and confidence in CHWs with household-level assignment. Positive relationships between CHWs in their households, community members, and health facility staff fostered health knowledge, individual agency, and personal resources for the community members to access health services. Community members’ personal resources of increased health knowledge, trust, gratitude, and social support improved social connectedness and subjective wellbeing. Areas to improve positive relationships include CHWs maintaining confidentiality and caring for pregnant women.

**Conclusion:**

Our study findings demonstrate that by building solid relationships as a community chosen, well informed, and household-level workforce, CHWs can develop positive relationships with communities and the health-care facility staff through building knowledge, trust, gratitude, and hope. Further work is needed in maintaining CHW confidentiality and new ways to approach culturally sensitive health areas.

## Background

Social connectedness is the practice of belonging and relatedness, centered on quantitative and qualitative social assessments and relationship strength, and is a vital determinant of wellbeing [1–3]. Social connectedness can be a powerful tool to utilize when planning public health interventions. Notable benefits of social connectedness are improved mental health, reduced depression, enhanced sense of belonging, improved health-seeking behavior, reduced stress, enhanced self-esteem, and cognition, leading to decreased morbidity and mortality [3–8]. Furthermore, evidence shows that people with decreased social connectedness have poorer mental and physical health outcomes than those with strong social interactions [4–7,9]. In places with poor technology availability and strong in-person community networks like Malawi, where only 48% of the population owns a cell phone or has access to the internet, physical means of interaction within the community is the primary mode for establishing social connectedness [10–13]. Successful community-based interventions depend on strong community and individual relationships, trust, gratitude, and good rapport with individuals and the community [14–21].

Community health workers (CHWs) are health-care workers who most commonly provide care delivery at the community or household level. Typically, they do not have professional training or degree in tertiary education but are locally trained in the context of the intervention and community they serve [21,22]. CHWs perform a critical role in connecting people within communities and providing screening, medical care, and linkage to vital health and social services [23]. Ample literature demonstrates that CHWs have contributed positively to improved health in rural and poor communities, for example, by improving treatment adherence in Human Immunodeficiency Virus (HIV) [22,24–29] and non-communicable disease (NCD) care [30].

Located in southwestern Malawi, the Neno District currently has an estimated 150,211 people, with most of the population working as subsistence farmers [31]. Neno is remote and rural, with no paved roads and only 3.7% of the population having access to electricity [31]. Starting in 2006, Partners In Health (PIH), known in Malawi as Abwenzi Pa Za Umoyo (APZU), a global non-governmental organization, began working in the Neno District. PIH works through an accompaniment model, employing close partnerships with the local government to build robust and equitable health systems for the most vulnerable communities [25,32]. PIH does not operate health facilities but integrates into the public facility operations of health ministries. Therefore, in partnership with the local Ministry of Health (MoH), a community health worker (CHW) cadre was introduced to extend health-care support to the household level.

Initially, CHWs in Neno District were utilized in a client-assigned accompaniment model focusing on supporting individual HIV and tuberculosis (T.B.) clients in psychosocial support, treatment adherence, and treatment response. Despite an overarching National Community Health Strategy [33,34] for community-based primary care in Malawi through the central Ministry of Health, CHWs often do not have clearly defined roles or consistent support at the lower levels due to a lack of resources. CHWs often respond to client-specific diseases that correspond to donor funding for vertical programming such as maternal child health [34–36]. However, in Neno District, there was recognition that this work was not responding to the disease burden. Additionally, we found that HIV and T.B. patients experienced stigma and discrimination as communities began associating a CHW visit with the presence of an HIV client. In 2016, to respond to the community needs and disease burden, the CHWs were transitioned to the household model in Neno District.

The household model of Neno District is a polyvalent public health model that assigns CHWs to each household in the district with increased and adjusted tasks. These include routine screening, referral, education, and disease management in eight key health areas – HIV, tuberculosis (T.B.), non-communicable diseases (NCDs), sexually transmitted infections (STIs), increasing the use of family planning among women of childbearing age, maternal and neonatal health, child health, and malnutrition. CHWs cover 20–40 households and are supervised by senior CHWs and site supervisors at each of the 14 catchment areas in the district. The CHWs work closely with the facility-based MoH community health-care workers – the health surveillance assistants (HSAs). The model positioned the community chosen CHWs as ‘foot soldiers’ to support the HSAs while receiving a volunteer stipend [35,37]. Due to the high workload of HSAs, the CHWs assist in integrated disease screening, linking clients to the essential health package at the community and health facility level, and raising community awareness on health, prompt care-seeking behaviors, and preventative practices such as hygiene [33].

We developed the household model to primarily improve the effectiveness of CHWs through the expansion of scope to respond to the burden of disease and the needs of the community. We aimed to maximize community education, engagement in services, and support through the model. The CHWs are selected by their communities, trained, and supported with connections to their health facility within each district’s catchment. During the household visit, CHWs conduct health education and promotion, screening with referrals as needed to the health facilities, and provide treatment adherence, monitoring, and psychosocial services for those with illness. Through continuous capacity building, we focused on building a well-organized network of knowledgeable and skilled CHWs capable of executing their tasks competently in a way that builds respect, trust, health knowledge, and positive relationships between communities and health facility staff.

This study utilizes aspects of Fredrickson’s broaden-and-build theory of positive emotions to explore deductive themes [38]. The theory suggests that ‘positive emotions such as joy, interest, contentment, and love broaden an individual’s momentary thought-action repertoire which promote discovery of novel and creative actions, ideas and social bonds.’ Positive emotions build an individual’s resources; ‘ranging from physical and intellectual to social and psychological resources.’ These personal resources can be drawn as a reserve by the individual throughout their life course to cope and thrive within their communities [38]. A study by Liao and Weng [39] further expanded on the broaden-and-build theory by demonstrating empirical correlations between social connectedness and subjective wellbeing. Results showed that changes in gratefulness (a positive emotion) predicted changes in social connectedness and the presence of meaning in life, which, in turn, enhanced personal wellbeing [39].

Studies have shown improvements in trust [14,15,20,40] and social connectedness [41] within communities since the introduction of CHWs [14,15,20,40,41]. However, there remain gaps in reported factors for CHWs enabling relationships and social connectedness between CHWs, communities, and health facilities from the user’s perspective, health-care providers, and the CHWs themselves. As part of a broader qualitative evaluation of the implementation of the household model, our study aims to qualitatively examine the factors that promote CHWs enabling relationships and social connectedness between communities and health facilities through the perspectives of the users, health-care providers, and the CHWs themselves.

## Methods

### Data collection and sampling

We conducted 12 focus group discussions (FGDs) (N = 140) with CHWs, facility health-care workers, and community members and 40 in-depth interviews (IDIs) (N = 40) with clients in four catchment areas of Neno District – Ligowe, Dambe, Chifunga, and Zalewa from October 2018 through March 2020. We chose these four groups of participants to represent diverse and varied viewpoints of the household model, all as actors within the program but with a unique insight as consumers, collaborators, and providers. FGDs were conducted with CHWs, community members, and health facility staff for collective viewpoints to reach saturation. Clients were individually interviewed in their homes for confidentiality and to create a safe environment without potential disclosure of health status. Both methods aimed to map themes to Fredrickson’s broaden-and-build theory of positive emotions for the role of the CHWs.

Out of the 14 catchment areas in the Neno District, we chose the four study sites as they have implemented the household model since its inception in 2016; thus, FGD and IDI participants would have a sustained experience with the model. FGDs involved 48 CHWs, 45 community stakeholders – general members, leaders, community-based organization members, and 47 facility health-care workers – clinical and nursing staff, facility-based CHWs, ancillary staff, and non-medical staff participated (Table 1). Clients for individual interviews were sampled purposively depending on their enrollment in chronic care programs (HIV, T.B., or NCD), pregnant women, and mothers of under five-year-old children without overlapping with participants of the community stakeholder focus groups. Most participants were between 25 and 39 years old, with relatively equal representation between males and females. Categories included experienced and newly employed CHWs and a broad range of participants from each type.

**Table 1.** Socio-demographic characteristics of participant categories

Variable	Community Health Workers N (%)	Community Members* N (%)	Facility healthcare workers N (%)	Clients N (%)	
Type of participation	Focus group discussion	Focus group discussion	Focus group discussion	Individual in-depth interviews	
Number of participants in each category	48 (27)	45 (25)	47 (26)	40 (22)	
Location					
Ligowe	12 (25)	11 (24)	11 (23)	10 (25)	
Dambe	12 (25)	11 (24)	12 (26)	10 (25)	
Chifunga	12 (25)	11 (24)	12 (26)	10 (25)	
Zalewa	12 (25)	12 (27)	12 (26)	10 (25)	
Age					
12- 24 years	0 (0)	2 (4)	3 (6)		8 (20)
25- 39 years	30 (63)	20 (44)	32 (68)		20 (50)
40-59 years	17 (35)	17 (38)	12 (26)		10 (25)
60 years and above	1 (2)	6 (13)	0 (0)		2 (5)
Gender					
Male	21 (44)	18 (40)	28 (60)		12 (30)
Female	27 (56)	27 (60)	19 (40)		28 (70)
CHW service (years)					
<5 years	18 (38)	-	-		-
>5 years	30 (63)	-	-		-
Community Members					
General	-	20 (44)	-		-
Leaders*	-	13 (29)	-		-
Community-Based Organizations**	-	12 (27)	-		-
Facility healthcare workers					
Clinicians	-	-	8 (17)		-
Nurses and midwives	-	-	7 (15)		-
Facility CHWs^‡^	-	-	8 (17)		-
Ancillary staff^†^	-	-	16 (34)		-
Non-medical staff^^^	-	-	8 (17)		-
Clients					
Chronic care programs (T.B., HIV, and NCD)	-	-	-		24 (60)
Pregnant women	-	-	-		8 (20)
Mothers of children <5 years old	-	-	-		8 (20)

*Included chiefs and village headmen

**Included community-based organization and village development committee members

^‡^Included Health Surveillance Assistants (HSAs) and Site Supervisors (SS)

^†^Included expert clients, health attendants, laboratory staff, counselors, and pharmacy staff

^Included data clerks, ground labor, and security guards

The FGDs and IDIs were part of a more extensive study evaluating the transition to the household model implementation in the Neno District over three years (2018–2020). There were no significant changes in the implementation of the model or CHW requirements during this period. Data were collected in three phases: 1) eight FGDs with CHWs and community stakeholders (N = 93) in October 2018; 2) forty in-depth interviews (N = 40) with clients enrolled in HIV, T.B., and NCDs chronic care programs in March–April 2019 and 3) four FGDs with facility health-care workers (N = 47) in March 2020. Participants were purposively sampled [42]. We preselected the sample size because it was more than the minimum commonly needed to achieve saturation, the point at which the data collection process no longer offers any new insights [43]. Researchers held frequent meetings to discuss responses to the study guides and the evolving findings concurrently with data collection. We reached saturation before completing all focus groups and interviews without the need to seek additional respondents.

## Data collection tools and procedures

In all the three qualitative phases, question guides included open-ended questions describing CHW’s role in the community that encourage dialogue and opening up. Probes for more detailed information followed these initial questions. The interview guides were developed in English, translated into Chichewa (local language), and pretested with CHWs before use, with translations adjusted before formal data collection. FGDs and IDIs were conducted and analyzed by a research fellow and trained research assistants hired by PIH/APZU without prior knowledge or work in implementing the household model or CHW work. Each FGD and IDI took approximately 2 hours and 45 minutes, respectively.

This study was approved by the National Health Science Research Committee (NHSRC) in Malawi with protocol number 1059, titled ‘*Lessons Learned from Monitoring and Evaluation of Community Health Initiatives in Neno District, Malawi*.’ Written informed consent was sought from all potential participants before their recruitment to the study. The study was conducted following the Declaration of Helsinki guidelines and regulations [44].

## Data analysis

FGDs and IDIs were conducted in Chichewa, transcribed verbatim, translated into English and then analysed thematically utilizing Fredrickson’s broaden-and-build theory [45]. The research fellow (first author MKN) transcribed data, and B.N. double-checked transcription by listening to all audio recordings and verifying the translation from Chichewa to English before loading them in Dedoose version 8.3.17 for data management.

The thematic analysis process started in the field through field notes taken by research assistants and MKN and debriefing immediately after every FGD and IDI exploring themes that came out recurrently. Other analytical processes involved familiarisation, coding, and mapping the data to Fredrickson’s broaden-and-build theory of positive emotions [38]. MKN familiarized with the data set through immersion by the repeated and active reading of transcripts [45]. To ensure the reliability and quality of coding and consistency, MKN, B.N., and E.C. independently read three systematically sampled transcripts line by line to deductively assign codes to similar concepts that repeatedly emerged from the data in line with study objectives [46]. Any identified differences in coding were discussed and resolved.

The first codebook was generated from these three transcripts through a consensus process by looking at commonalities and differences [46] with the broaden-and-build theory [38]. MKN then coded the rest of the transcripts, with feedback from B.N. and E.C., deleted repeated codes, and added new ones until a final codebook was created. The final codebook was agreed upon by the joint consensus of all authors [46]. We identified relationships between codes with repeatedly recognized codes merged, and themes and sub-themes were generated from these codes. We chose an exemplar extract quotes for each theme and sub-theme summarizing the main points [45,46]. We ensured triangulation of views presented by collecting data from four participant categories. Moreover, member checking between categories and the use of the two data collection methods (FGDs and IDIs) allowed for individual and collective viewpoints triangulation.

## Results

**Figure 1.**
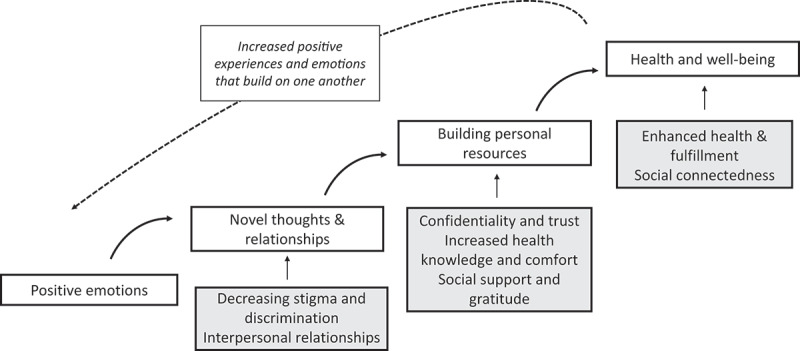
Mapping of deductive themes to Fredrickson’s broaden and build theory.

The findings of this study were deductively identified around common themes linked to Fredrickson’s broaden-and-build theory of positive emotions, including; i) novel thoughts and relationships, ii) building personal resources, and iii) enhanced health and wellbeing (Figure 1) [38]. Themes and functional elements were either expressed in the stakeholder category and validated by more than one category or expressed within multiple individual interviews with minor expression differences.

### Novel thoughts and relationships

#### Stigma and discrimination

The transition from an individualized client-based model to the polyvalent expansion of care with the household model decreased stigma and discrimination by enhancing social interactions and relationships between the CHWs and communities. Participants mainly in the community member and client categories noted that in the initial client disease-based model, communities associated a CHW visit with the presence of an HIV-positive or T.B. client, which inadvertently exposed a client’s confidential disease status leading to stigma and discrimination. Representative quotes in both categories reflected the stigma:

This arrangement is good. At first people could say that if the CHW visits you it means you have HIV. Now that everyone is visited, the relationship has been well cemented because people do not have an idea of who is suffering from what hence we interact freely because of this new arrangement. **Man on Antiretroviral Therapy and Tuberculosis treatment – IDI-Zalewa**

When the CHW was visiting a single person in the household, it was like the husband and the wife were doing things separately but now we are doing things together as a household since we are all visited. The relationship with our health worker is good as well. Now men are even able to go to the ANC with their wives. **Female Community Stakeholder-FGD-Chifunga**

Participants in all the clients, community members, and facility staff reported that the household model with impartial visits to each family in the community decreased stigma among community members. The CHW health education and promotion also led to open communication with friends and relatives. Quotes from a client and a Health Surveillance Assistant from the health facility exemplified this:

The CHW treats us equally. Does not differentiate that this one is HIV positive and this one is not. That makes us follow her example hence there is no stigma and discrimination here. Sometimes she will even teach my relatives to treat me equally despite my condition which also contributed to the good care that my relatives give me. **Woman on Antiretroviral Therapy for more than one year- IDI-Zalewa**

Previously, people were discriminating each other, but with the messages that CHWs have disseminated, people have accepted that everyone can get sick and they are able to live together with their relatives. **A Health Surveillance Assistant –FGD- Ligowe**

Despite the positive perception change for clients in chronic disease programs, the transition to the household model has not eliminated stigma and bias in pregnancy, which are longstanding community beliefs. As mentioned by CHWs and pregnant clients, most pregnant women are highly secretive and unwilling to disclose any information that will indicate that they are pregnant.

Screening for pregnancy is really difficult. Sometimes you can tell that someone is pregnant but they do not accept to say they are pregnant in fear of being referred to antenatal and also thinking that they will lose the pregnancy. **Female CHW-FGD-Chifunga**

… about pregnancy screening, you feel like maybe she [the CHW] has a wrong motive behind [wants to bewitch you maybe] so it is difficult to be very open. Sometimes they will come early in the morning and they ask you about menses … Sometimes I lie just to do away with her. **Adolescent pregnant woman – IDI-Chifunga**

Furthermore, the CHW relationships and education have not significantly decreased the blame and stigma of adolescent pregnancy, as noted by pregnant adolescents among the client participants:

My parents are very harsh on me such that they shout at me every day saying I should refund the money that they have been spending on my school fees and pocket money … They told me that I should be responsible for all the expenses on this pregnancy. Up to now I am fetching for the labor materials on my own. **Adolescent pregnant woman –IDI- Dambe**

#### Interpersonal relationships

Participants in all categories heralded CHWs for unprejudiced interfaces in their communities with a positive outlook and message, which led many individuals in the community to emulate them. Participants from FGDs and IDIs reported improved interaction between themselves, their families, friends, and the community with the transition to the household model. This point was reflected in quotes from a CHW and community member:

There is no more gossiping about each other’s problem now. People are able to assist each other whilst previously people were not helping each other for fear of getting the disease. We have been teaching them that some of these diseases are inherited/genetically acquired [and therefore not contagious] **Male CHW-FGD-Ligowe**

I don’t think there is a problem if a CHW finds one spouse only. If we say the CHW should wait for both people to be present it will create another problem. For instance, I was sick and my wife was not at home but the CHW visited me and counselled me, escorted me to the hospital and I am now on T.B. treatment. If she was to wait for my wife to be present, I could have been dead by now. **Male Community Stakeholder-FGD-Zalewa**

Furthermore, respondents from the client and community stakeholder categories reported that CHWs often share messages of hope for those who have lost hope, love for those who feel lonely, and peace for those troubled in their hearts, as explained below:

CHWs have enhanced relationships between chiefs and their people as they link them through different forums. They encourage us to love each other during problems and otherwise. CHWs have helped us to coordinate with our neighbors, for instance, when someone is sick we organize to go to the hospital as a team and then the CHW follows us later. **Female Community Stakeholder- FGD -Chifunga**

### Building personal resources

#### Confidentiality and trust

Clients, facility health-care workers, and community members agreed that trusting the advice, psychosocial support, and referral of a CHW with confidentiality of client information is one of the most critical aspects of the household model program. Participants perceived that this act improves outcomes and enhances confidence in the CHW’s ability to provide screening and care, recognizing that communities would have higher morbidity and mortality rates if no CHWs worked in their areas. This aspect was exemplified through client and health-care worker responses:

Mmmmm I cannot hide anything from him. If I hide then I can die. Even if it is about my marriage, I would still let him know. Because he is like my doctor so I am free to tell him whatever problem I can encounter and he assists accordingly. **Man with Non-Communicable Disease –IDI-Ligowe**

Yes, they are a very trusted cadre. We have few staff at facility level so we rely on them to link us to clients. They are like eyes, ears, hands, legs and a voice of those that work at facility level. Also, they are very good at keeping secrets in regard to patient information hence we trust them. **Female nurse –FGD- Ligowe**

While most participants expressed confidence in CHWs and their work, a few participants in the community stakeholder category mentioned that not every CHW builds trust and confidence in the community. This point was noted due to the personal faults of the CHW as reflected below:

We had one who could reveal secrets when drunk but was counselled and it has changed now … so you cannot confide in someone who doesn’t keep secrets, it’s double impossible. **Male Community Stakeholder- FGD-Ligowe**

Moreover, HSAs in the health facility category supported that most CHWs are trustworthy. HSAs perceive that even though a smaller number of CHWs might not keep secrets, most CHWs take their role seriously with respect for their clients. Instead, they shared a deeply rooted community expectation to hold personal information close despite strong CHW relationships.

Some do not trust their CHW not because they did something wrong, but they just don’t like sharing their personal information. Such people will even discourage others to confide in CHWs. So it’s just the mindset, not that they don’t keep secrets. We cannot rule out though that some don’t keep secrets, but it’s a very small percentage. **Health Surveillance Assistant –FGD- Dambe**

#### Increased health knowledge and comfort

Participants mainly in the community stakeholder category noted that expanding services to more disease areas and households provides educational opportunities to clients and communities. They expressed that CHWs could share health messages and knowledge promoting health and wellbeing through this role. For example, with prevention and treatment for malnutrition:

We thought that the only way of helping a malnourished child is by feeding him/her ready-to-use therapeutic food but the CHWs have taught us to use the readily available food, which we have in our communities … Malnutrition has decreased because of the advice they gave us and we are able to feed our children the locally available food. **Female Community Stakeholder-FGD-Dambe**

Furthermore, community leaders felt that with CHWs in the communities, there had been increased health-seeking behaviors such as the use of maternal waiting homes at the facility. As community leaders noted:

Without CHWs, we could have high mortality rate, more especially on maternal deaths. CHWs encourage women to go to the waiting shelter when their delivery time is near. **Chief –Community Stakeholder-FGD-Chifunga**

Most people in this village had poor health seeking behaviour [before CHWs were in place]. Now without CHWs, the community would have been full of sick people hence we could have more deaths. CHWs are really assisting. Sometimes we relax without knowing that we have a problem. **Community leader-Community Stakeholder-FGD-Zalewa**

Lastly, participants in all categories noted that CHWs often refer screened clients with any positive symptoms to the facility, accompany clients to clinic visits, and track and return clients to care. Participants shared that these actions enhance health-seeking behavior and help clients feel gratitude for an advocate and empowerment in their health and wellbeing.

… Yes they link clients to care. For instance, last week there was a client who came here Monday. He got sick on Friday but the relatives were just keeping him. When the CHW .knew that there is a client at that household, he went there and brought the client here. **Male Clinician –FGD- Dambe**

… it took the CHW to link me to the hospital when I was too sick … it made me feel that I am also important and I started interacting with other people since I was encouraged. So without the CHW we could not know which direction to take. **Woman on Antiretroviral Therapy for more than one year-IDI- Zalewa**

#### Social support and gratitude

As CHWs conduct home visits, they assess the household’s social and economic situation and link the vulnerable to available social support programs in the district. These include a program with PIH/APZU and the social welfare office of the district. Participants reported that these efforts offered hope for community members in poverty and built gratitude for the CHW.

I was very sick and nearly gone. My house was dilapidated such that people could not drink water at my house. When I gained strength and started walking, sometimes I would feel thirsty and when I ask people to give me water to drink, they would use some old cups that they do not use … When Partners In Health built a house for me, people started coming to see me so I feel like this house enabled people to start interacting with me. I guess they saw that I am now important. **Woman on Antiretroviral Therapy for more than one year –IDI- Chifunga**

#### Enhanced health and well-being

Health-seeking behavior and CHW accompaniment build personal resources of gratitude, resilience, confidence, and positive relationships. These actions improve the individual’s overall feelings of health and wellbeing.

Without CHWs, we could have a lot of sick people who cannot walk by themselves in this community as it was before. CHWs have empowered people to have confidence in their services hence a lot of people seek care at an early stage. This has been possible through good relationships among community members, CHWs and health facility staff facilitated by CHWs. **Male Community Stakeholder-FGD-Chifunga**

Without CHWs, I could have been dead by now. The positive interactions between me and my CHW, the CHW and my relatives, and the CHW and people working at the hospital gave me trust in the CHW services and eventually improved my health seeking behavior. At first, I thought I was bewitched and I wasted more time visiting witch doctors. **Woman on Antiretroviral Therapy for more than one year –IDI- Chifunga**

## Discussion

This exploratory study explores and discusses the aspects of the CHW role that promote social connectedness from the perspective of the CHWs, community stakeholders, clients, and facility health-care workers to improve primary care delivery and promote health. The findings highlight that CHWs can help build new thoughts, relationships, personal resources, and promote health and social connectedness. Through the lens of Fredrickson’s broaden-and-build theory of positive emotions, the building of knowledge and relationships, in turn, builds feelings of improved health, wellbeing, and social connectedness in individuals and communities [38].

Through the broaden-and-build theory within the clients, community members, and facility health-care workers, we found that CHWs immediately garner trust and a sense of comfort in providing health education and services to community members. Similarly, the CHW participants shared feelings about meeting individual and community needs through their roles. Likewise, prior CHW studies have demonstrated that when CHWs are chosen and capacitated within their communities, it builds trust in their services and abilities with individuals in their community [15,47–49]. Furthermore, through the polyvalent model, CHWs could decrease stigma and discrimination by not focusing on patients with HIV or T.B. and equal treatment of all individuals and households with the provision of health education and promotion. These actions have forged strong individual and community trust and positive emotions, which has led to improved interpersonal relationships not just with CHWs but with families, friends, and the community. As one participant shared that CHWs have encouraged community actions to help each other with love and care. Similarly, an ethnographic study in India [15] showed that CHWs perceived their work not just as providing health services but also as ‘teamwork” and building trust with the community. Additional quantitative and qualitative studies, mainly from Asia and Africa [47,48], show that CHWs can positively influence individuals when programs ensure frequent visits with accompaniment, trust, communication, and friendship in addition to health services.

Despite many CHWs building strong linkages with the community, some participating CHWs and several pregnant clients reported continued secrecy, bias, and stigma in early pregnancy for all women. The culture in rural Malawi not to report a pregnancy at an early stage is due to a fear of witchcraft or evil wishes leading to a miscarriage or poor outcomes [50]. This belief is a longstanding norm that even health-care providers have struggled with and was reflected by facility health-care workers in their FGDs. CHWs and pregnant clients noted that CHWs have similar struggles with detecting early pregnancy for robust antenatal care leading to improved pregnancy outcomes. This challenge of early pregnancy reporting also has been identified for CHWs in Tanzania [51], Malawi [36], and Uganda [52]. They identified similar concerns of losing the pregnancy and witchcraft, not wanting to tell a young female CHW without prior experience, and the expectation that a pregnant woman must first confirm the pregnancy at the facility before telling a CHW.

CHWs and adolescent pregnant women participants shared that CHWs have had little effect on the stigma and social isolation of pregnant adolescent women in the Neno district. Adolescent women are subject to rejection with pregnancy due to assumptions of promiscuity and lack of abstinence on the girl’s part [53,54]. As a result, the young woman’s family is often shamed and discriminated against in the community, especially if the pregnancy leads to the girl dropping out of school or getting married before 18 years old [55]. Despite several CHW programs working to decrease discrimination against adolescent pregnancy or incidence of adolescent pregnancy, a systematic review in 2016 showed that CHWs have very little influence in preventing adolescent pregnancy or the challenges pregnant youth face with many cultural and social implications [56]. As shown in other studies, we must aim to find facilitating factors for early pregnancy detection, such as husband involvement in screening and birth-preparedness activities, to enrich connections between the CHW, the community, and pregnant women [36,51,52]. More efforts are required to invest in multidisciplinary programming to influence the cultural cycle, including education, youth-friendly health services, and building trust in youth and their parents to prevent and support adolescent pregnancy. Studies have found that CHWs can play an essential preventative, connective, and supportive role in this work [47,49,56,57].

Building on the trust and interpersonal relationships through the household model, all four participant groups shared that CHWs assist in building personal resources of increased health knowledge and support for seeking health services through provision of physical and psychosocial assistance. Clients, community members, and facility staff shared that the core aspect of building these resources was regular visits, regardless of health status, building the relationship. Through these visits, CHWs were able to help them understand their health and treatment and prevention of disease with presumptive screening and referrals to clinics to keep their clients’ confidentiality. Additionally, community members shared that CHWs were able to link vulnerable clients and households to social support and advocate on their behalf. Other studies have shown or argued that if CHWs can build personal resources for their clients, it results in increased health understanding and seeking behaviors with trust playing a central role [15,47,49]. Conversely, as seen in one example in this study, the therapeutic and educational relationship suffers if community members do not trust the CHW. The break in trust can be seen with a lack of confidentiality from CHWs, as seen in one example of our study and other studies [14,58,59].

Through the broaden-and-build theory of positive emotions, we can understand how positive emotions and new ideas within the communities built on improved trust and interpersonal relationships could expand health knowledge and community support for themselves and health. Outcomes include improved primary health-care interactions, care-seeking behaviors, and better experiences for individuals and communities [21,41]. There is additional work to be done from challenges identified in pregnancy, likely through community dialogue and participatory action research to understand better, counsel, and care for pregnant women, especially adolescents. These findings have implications for not just the CHW program in Neno District but throughout Malawi and beyond. Through inclusive and horizontal primary care with CHWs in well-defined supported roles, the foundational relationship of trust and improved personal resources lead to deepened social connections and an enhanced feeling of overall wellbeing.

Given our qualitative methods within a single rural district, these results may not be broadly generalized outside our local context or household model implemented along with a longstanding relationship with the Neno District. However, these methods and populations are well suited to illustrate nuanced social connectedness through the broaden-and-build theory. Social connectedness is not easily understood by utilizing quantitative measures alone, and these methods allow the encompassing of multifaceted and multisectoral relationships and community connections. Even with the limitations, we believe that this investigation provides an illustrative portrait of how we can build social connectedness through a comprehensive community health worker program through positive thoughts, relationships, and personal resources.

## Conclusion

Our study demonstrates that polyvalent CHWs can build personal resources of trust, gratitude, and hope within the communities, fostering positive interpersonal relationships between themselves, communities and health facility staff. These connections minimized stigma, expanded care, and treatment and increased the ability of CHWs to promote novel thoughts, relationships, and personal resources that lead to social connectedness and feelings of wellbeing. Further work will include developing new ways to approach culturally sensitive areas to increase social connections and positive health experiences.

## Author contributions

MKN, B.N., and E.C. conceptualized and designed the study. MKN collected data, analyzed data, and drafted the manuscript with assistance from B.N. and E.C. B.N. and E.C. also reviewed the transcripts and the codebook. E.C., B.N., FM, H.M., and M.A. reviewed the manuscript, provided input, and suggested additions and changes. All authors read and approved the final manuscript.

## Paper context

Community Health Workers (CHWs) play vital roles in providing community-level primary health services. Further understanding of the drivers of positive CHW interactions within communities with enhanced care seeking behavior and wellbeing is needed. In a household-level program, we found that the CHW role fortifies positive relationships with increased individual agency, which enhances feelings of social connectedness and improved wellbeing. Further work is needed in reinforcing confidentiality and carefully approaching culturally sensitive topics.
